# Prevalence and Risk Factors for Hand-Grip-Determined Dynapenia in the Korean Population: A Cross-Sectional Study

**DOI:** 10.3390/sports12070187

**Published:** 2024-07-09

**Authors:** Do-Youn Lee

**Affiliations:** Department of Physical Therapy, College of Rehabilitation Science, Daegu University, Gyeongsan 38453, Republic of Korea; triptoyoun@kookmin.ac.kr; Tel.: +82-02-910-5540

**Keywords:** dynapenia, prevalence, risk factors

## Abstract

Dynapenia refers to muscle weakness related to aging and is defined as a loss of muscle strength associated with muscle quality. The purpose of this study is to identify the prevalence of dynapenia and associated risk factors by gender and age in domestic adults and to provide customized basic data for the prevention of dynapenia through its management. Based on the data from 2014 to 2019 of the Korea National Health and Nutrition Survey, 20,950 adults over the age of 20 who participated in grip strength tests and health surveys were selected as participants. Factors related to dynapenia were analyzed using complex sample multilogistic regression analysis. The prevalence of dynapenia in domestic adults was 6.3%, 4.1% in men, and 8.7% in women. Prevalence in the second decade was 5.3%, in the third decade was 3.2%, in the fourth decade was 3.3%, in the fifth decade was 4.8%, in the sixth decade was 8.9%, and was 24.6% for participants in their seventh decade or beyond. Among the factors related to dynapenia, age, BMI, and alcohol status were common in both men and women; education level, HDL-C, and resistance exercise were common in men; and high blood pressure, high blood sugar, and aerobic exercise were common in women. Our findings indicate that several factors are associated with dynapenia, which should be considered as potential targets for interventions focused on both individual and public health measures.

## 1. Introduction

Dynapenia is characterized by an age-related decrease in muscle strength, which is associated with muscle quality and is defined as a loss of muscle strength and power [1]. Dynapenia poses a significant challenge to the health and well-being of the older adult population worldwide [2]. The gradual loss of muscle mass and strength with aging not only impairs physical performance but also increases susceptibility to various health comorbidities [3,4,5,6].

Dynapenia differs somewhat from sarcopenia, which is defined as a decrease in muscle or lean body mass [1,7]. The decline in muscle strength occurs at a much faster rate than that in muscle mass, indicating that the pathophysiologies of sarcopenia and dynapenia may differ [8]. Moreover, muscle strength measurement is a key step in diagnosing sarcopenia, and treatment for sarcopenia is recommended regardless of whether there is a decrease in muscle mass [9].

Dynapenia is measured through grip strength, which serves as a single indicator of muscle strength and is primarily utilized to diagnose dynapenia and sarcopenia [10]. Grip strength provides a convenient and easily measurable parameter for assessing maximal voluntary muscle strength [11]. Grip strength influences functional aspects of life in various domains and contributes to better performance in activities of daily living [12].

Understanding the sex- and age-specific risk factors for dynapenia is essential for several reasons. First, biological differences between males and females can influence muscle composition and function, potentially leading to varying susceptibility to muscle strength conditions. Second, the aging process exacerbates muscle loss, with older individuals experiencing more rapid decreases in muscle strength than younger individuals. Third, decreased muscle strength affects physical, psychological, and cognitive domains across all ages and sexes [13,14], with potential differences based on sex and age.

Previous studies related to dynapenia have mainly investigated risk factors and prevalence in elderly individuals and have not distinguished factors affecting muscle strength in elderly individuals, even though they may differ by sex and age [15,16,17]. To reduce the prevalence of dynapenia, it is essential to control for relevant factors, and research analyzing risk factors for this condition is crucial. Therefore, exploring the interaction between sex and age-specific risk factors for dynapenia can provide valuable insights into its underlying mechanisms and help inform targeted interventions for prevention and management. This study aimed to identify these specific risk factors for dynapenia, defined by grip strength, by examining socio-demographic, lifestyle, and physiological variables. The purpose of this study was to reveal how these factors differentially affect dynapenia across gender and age groups, ultimately providing evidence-based recommendations for customized interventions based on comprehensive data analysis.

The detailed purpose of this study is as follows. First, the prevalence of dynapenia stratified by sex and age based on grip strength was examined. Second, the socio-demographic characteristics and health-related factors of dynapenia according to sex were analyzed. Third, the risk factors for dynapenia according to sex were identified.

## 2. Materials and Methods

### 2.1. Data Source and Sampling

**Figure 1:**
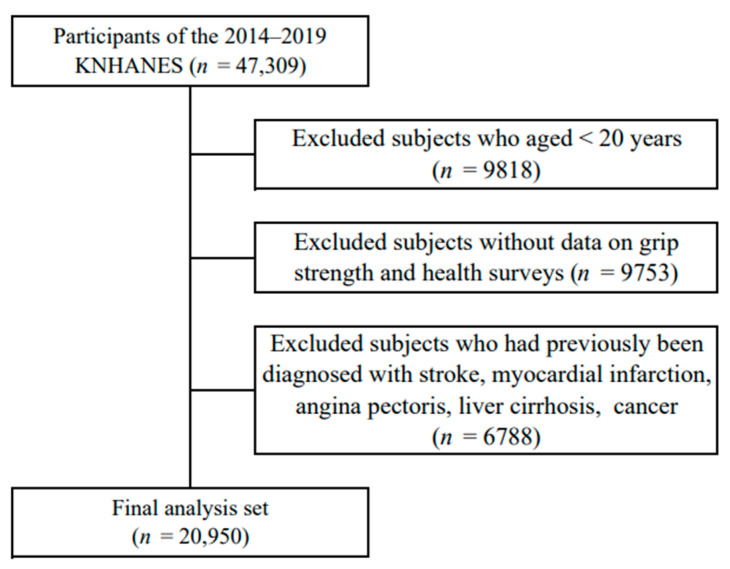
Selection of participants from the Korea National Health and Nutrition Examination Survey 2014–2019.

This study used data from the Korea national health and nutrition examination survey (KNHANES) 2014–2019, which was conducted by the Korean Centers for Disease Control and Prevention (KCDC). Those who responded to both the examination survey and the health survey among adults aged 20 years or older whose grip strength was measured were included in this study. Among the 47,309 individuals who participated in the KNHANES, 9818 were under the age of 20 years, 9753 did not complete the grip strength test or health survey, and 6788 had severe diseases such as stroke, myocardial infarction, or angina. Finally, 20,950 participants were selected (Figure 1). KNHANES followed the standards outlined in the Declaration of Helsinki. All participants signed an informed consent form. The KCDC’s institutional review board approved the protocol.

### 2.2. Socio-Demographic Factors

Demographic and sociological variables such as sex, age, education level, marital status, and individual income level were recorded. Age was classified as 20s, 30s, 40s, 50s, 60s, and 70s or older. Education levels were classified as Low or High based on high school graduation. Marital status was determined by whether the respondent lived with their current spouse. For individual income level, the average monthly individual income was divided by the quartile.

### 2.3. Health and Disease-Related Factors

The health and disease-related variables measured included height, weight, body mass index (BMI), blood pressure, fasting glucose, triglycerides, high-density lipoprotein cholesterol (HDL-C), waist circumference, smoking and drinking status, and aerobic and resistance exercise. The BMI was calculated by dividing body weight (kg) by the square of height (m^2^). The participants were classified as underweight, normal, overweight, or obese. Blood pressure was measured using a mercury spherometer, and after 5 min of stability with a cuff that fit the arm circumference, blood pressure was measured three times at 30 s intervals. Hypertension was defined as a systolic blood pressure greater than 130 mmHg, diastolic blood pressure greater than 85 mmHg, or current use of antihypertensive medications. Blood samples were taken with an empty stomach for more than 8 h and analyzed within 24 h on a Hitachi Automatic Analyzer 7600 (Hitachi, Tokyo, Japan). Diabetes (hyperglycemia) was defined as a fasting glucose level ≥100 mg/dL or the use of diabetes medications. Hypertriglyceridemia was classified as a tri-glyceride concentration higher than 150 mg/dL. Low HDL-C was defined as a level less than 40 mg/dL in men and less than 50 mg/dL in women, while abdominal obesity was defined as a level greater than 90 cm in men and greater than 85 cm in women based on waist circumference (WC) [18].

In terms of smoking status, when asked about current smoking status, “daily smoking” and “occasional smoking” were classified as current smoking, “I smoked in the past, but I did not smoke in the present” was classified as past smoking, and “I never smoked” was classified as non-smoking. Current drinking was defined as “more than once a month”, while nondrinking was defined as “less than once a month” and “I did not drink at all in the past year” [19].

Aerobic exercise was calculated using the following walking time. The number of days the subject walked ≥10 min at a time for the past week was recorded. Walking was measured by total walking time per week (TWT), which was calculated as follows: TWT = walking days (days/week) × walking minutes (minutes/day). The frequency of resistance exercise was determined by participants’ responses to the question, “How many times do you perform resistance exercise (push-ups, sit-ups, lifting dumbbells or barbells) a week?” If there was no resistance exercise at all, the participants were divided into two groups: medium intensity for 1–3 days of resistance exercise and high intensity for more than 4 days [20].

### 2.4. Dynapenia Definitions

Grip strength was measured with a digital hand dynamometer (T.K.K 5401; Takei, Tokyo, Japan). The participants held the hand dynamometer as hard as possible for 3 s, three times per hand, while the patient stood with their forearm extended laterally at the thigh level. Each measurement was separated by a minimum 60 s rest interval. Dynapenia was defined as the maximal measured grip strength among the six measurements (<28 kg for men, <18 kg for women) [21].

### 2.5. Data Analysis

The SPSS 28.0 (IBM, Armonk, NY, USA) program was used for data analysis in this study, and the statistical significance level was set at 0.05. KNHANES used complex weighting and stratification methods to be representative of the Korean population [4]. The specific analysis method is as follows.

First, the differences in characteristics between the dynapenia and the normal group were analyzed by *t*-test and complex sample chi-square test (χ^2^-test), and variance estimation was compared using standard errors (SE). Second, complex sample multiple logistic regression analysis was used to analyze the risk factors affecting dynapenia, and the statistics were expressed as odds ratio (OR) and 95% confidence interval (CI).

## 3. Results

### 3.1. Dynapenia Prevalence and Socio-Demographic Characteristics

**Figure 2:**
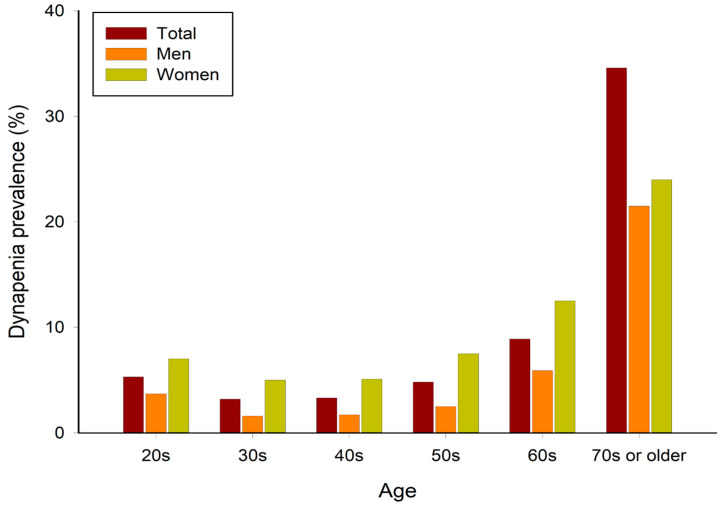
Prevalence of dynapenia according to age.

The prevalence of dynapenia by age group is shown in Table 1 and Figure 2. The overall prevalence of dynapenia among adults in their 20s and older in Korea was 6.3%, and the average grip strength was 33.59 ± 0.11. When classified by sex, the prevalence and grip strength were 4.1% and 41.60 ± 0.11 in men and 8.7% and 24.69 ± 0.07 in women, respectively. The prevalence of dynapenia was the lowest in those in their 30s, and after that, it tended to increase with age, and there was a rapid increase in those in their 70s and older.

Socio-demographic characteristics according to gender and dynapenia are presented in Table 2. Significant differences were found in age, education level, marital status, and individual income between men and women.

### 3.2. Health and Disease-Related Characteristics

The health and disease-related characteristics according to the sex and dynapenia of the subjects of this study are presented in Table 3. In men, significant differences were found in all variables except fasting glucose, HDL-C, and aerobic exercise. In women, significant differences were found in all variables except for the BMI index. BMI was 22.88 ± 0.14 in the dynapenia group and 22.89 ± 0.04 in the normal group; there was no significant difference, but when BMI was categorized, there was a significant difference.

### 3.3. Factors for Hand-Grip-Determined Dynapenia

Table 4 and Table 5 show the related factors of dynapenia occurrence. Univariate logistic regression analysis revealed that the risk factors of dynapenia in men were significantly different in all variables except fasting glucose and aerobic exercise. However, the risk factors identified in the final multivariate logistic regression analysis after correcting the variables that may affect were age, education level, BMI, HDL-C, alcohol status, and resistance exercise in Table 4. On the other hand, the final risk factors for dynapenia in women were age, BMI, hypertension, hyperglycemia, alcohol status and aerobic exercise, as shown in Table 5.

## 4. Discussion

This study was conducted to provide healthcare strategies and customized interventions for dynapenia by identifying the prevalence and risk factors for dynapenia according to sex and age. According to the results of this study, the prevalence of dynapenia in Korea was 6.3% overall, 4.1% in men, and 8.7% in women. The prevalence by age was the lowest in the 30s, and from there, the prevalence tended to increase with age.

In the present study, age, BMI, and alcohol status were found to be common risk factors for dynapenia in both men and women. The risk of dynapenia was significantly greater among those in their 70s and older than in those in their 20s, in both men and women. The grip strength shows a common pattern of peaking in the early 20s to 30s and continuously decreasing from middle to old age after the 40s [22,23,24]. In this study, the average grip strength in the 30s was the highest at 35.53 ± 0.19 and gradually decreased, with a sharply low grip strength of 25.53 ± 0.25 in those in their 70s and older. In addition, according to several previous studies, the average grip strength of men decreases more rapidly with age, and the gender difference tends to narrow with increasing age [23,24,25].

In men, the risk of dynapenia was significantly greater in the low-weight group than in the normal BMI group, and no significant difference was found in the overweight and obese groups. Among women, it was significantly higher in the low-weight group compared to the normal group and significantly lower in the overweight and obese groups, respectively. In both men and women, the higher the BMI in all age groups, the stronger the grip strength. In particular, in their 40s, the greater the body fat percentage was, the stronger the grip power [26]. In another study, there was an inverse correlation between BMI and grip strength in adults over 25 years of age [27], and the results of these preceding studies are similar to those of this study. This phenomenon may have occurred because the group with a lower BMI may have relatively chronic malnutrition, and a higher BMI reflects higher levels of muscle mass and fat mass [28,29]. In particular, this effect is thought to have resulted from the fact that it may have more influence on elderly people [30].

Alcohol status was found to be a risk factor for both men and women. In dynapenia, 54.8% of the current drinkers were men, and 52.5% were women; these percentages were significantly lower than the 86.1% and 72.6% of nondynapenia drinkers, respectively. In a previous study, a longitudinal study reported that the more alcohol consumed, the greater the decrease in grip strength [31]. In contrast, some studies have suggested that drinking is not associated with a decrease in grip strength or muscle mass [32]. Similar to the results of this study, one study found that moderate drinking (1–35 cups per week) reduced mobility and arm function limitations in the elderly [33]. Another study suggested that alcohol consumption may play a role in protecting aging-related muscle loss [34]. As such, the results of studies on the effect of drinking on muscle strength and muscle mass are mixed, and follow-up studies are needed to determine the associations and causal relationships.

According to the results of this study, education level, resistance exercise, and HDL-C were found to be associated factors for men. The risk of dynapenia was 1.512 times greater for individuals with a low education level than those with a high education level. A previous study revealed that when all socio-demographic and health-related covariates were kept constant, the higher the education level was, the healthier the diet and the greater the likelihood of participating in physical activity [35]. In another study, physical activity was shown to be a major mediator of the relationship between education and grip strength, and this mediating effect was such that the greater the education level was, the greater the number of participants in physical activity and the greater the participants’ grip strength [36]. The results of these preceding studies are consistent with the higher risk of dynapenia in participants with low education levels in this study.

The prevalence of dynapenia was 2.386 times greater in never-intensity exercise and 1.208 times greater in mid-intensity exercise than in high-intensity resistance exercise in men. Resistance exercise is the most effective way to improve muscle strength [37]. In particular, high-strength resistance exercise effectively improves muscle strength in elderly individuals [38]. Therefore, resistance exercise is essential as a strategy to prevent overall muscle weakness.

Patients with low HDL-C levels had a 1.299-fold greater risk of dynapenia than normal males. One prospective study revealed an independent association between muscle and HDL-C, with a 14% increase in HDL-C concentration and an increase in muscle mass resulting from the application of 8 weeks of resistance exercise in 25 young males [39]. In addition, a decrease in grip strength was associated with a decrease in HDL-C [40].

According to the results of this study, hypertension, diabetes, and aerobic exercise were found to be risk factors for dynapenia in women. In several previous studies, grip strength has been referred to as a risk index for type 2 diabetes and hypertension [41,42,43]. There is a significant association between grip strength and underlying diseases such as hypertension and diabetes. The physiological mechanisms underlying this association are not well known, but a decrease in muscle strength may occur due to increased oxidative stress and chronic inflammation [43]. In addition, the association between muscle strength and diabetes may occur due to the induction of insulin resistance in skeletal muscle [44]. One previous study revealed that elderly women with diabetes who regularly performed aerobic exercise through walking for 10 weeks had increased grip strength [45]. Another study revealed that aerobic exercise significantly increased bone metabolism and grip strength in patients with type 2 diabetes [46]. In addition, aerobic exercise significantly increased grip strength in coronary artery disease patients [47]. Furthermore, long-term aerobic exercise alleviated the muscle loss associated with aging and improved cardiopulmonary and metabolic functions [48]. As such, hypertension, diabetes, and aerobic exercise are thought to be factors that can affect dynapenia in combination.

This study has several limitations. First, this was a cross-sectional study that con-firmed the prevalence and risk factors for dynapenia at the time of the investigation, so there is a limit to explaining the causal relationship. Therefore, it is necessary to confirm this causal relationship through cohort studies in the future. Second, the grip strength values used in this study were not used to investigate how much factors such as height and race affect the difference in grip strength between regions. Third, in the KNHANES, the small number of severe dynapenia patients who did not participate may have influenced the outcome analysis. However, since the KNHANES data were obtained from the national population, it is thought that the disturbing factors for a small number of people did not significantly affect the results. Fourth, since the grip strength used in this study was based on the highest value, it does not contain information on left and right asymmetry.

## 5. Conclusions

This study was conducted to provide evidence-based recommendations for customized interventions by identifying the prevalence and related factors of dynapenia by sex and age in Korean adults. According to the results of this study, the prevalence of dynapenia in adults over 20 years of age in Korea was 6.3%, and when classified by sex, it was 4.1% in men and 8.7% in women. By age, 5.3% were in their 20s, 3.2% were in their 30s, 3.3% were in their 40s, 4.8% were in their 50s, 8.9% were in their 60s, and 34.6% were in their 70s or older. The factors related to dynapenia were age, BMI, alcohol consumption status (both men and women), education level, HDL-C, resistance exercise (men), hyper-tension, hyperglycemia, and aerobic exercise (women). Therefore, to lower the prevalence of dynapenia and prevent functional limitations and disabilities in the future, early interventions involving identifying subjects with risk factors are needed.

## Figures and Tables

**Table 1:** Dynapenia prevalence and grip strength according to age.

Variables		Total	20s	30s	40s	50s	60s	70s or Older
Total	Prevalence	6.3	5.3	3.2	3.3	4.8	8.9	34.6
	GS (M ± SE)	33.59 ± 0.11	33.54 ± 0.23	35.53 ± 0.19	34.85 ± 0.17	33.48 ± 0.18	31.10 ± 0.18	25.53 ± 0.25
Men	Prevalence	4.1	3.7	1.6	1.7	2.5	5.9	21.5
	GS (M ± SE)	41.60 ± 0.11	41.54 ± 0.23	44.50 ± 0.21	43.29 ± 0.19	41.25 ± 0.17	37.70 ± 0.18	34.06 ± 0.25
Women	Prevalence	8.7	7	5	5.1	7.5	12.5	24
	GS (M ± SE)	4	24.61 ± 0.15	25.87 ± 0.12	25.77 ± 0.11	24.56 ± 0.11	23.06 ± 0.14	21.77 ± 0.17

GS, grip strength; M ± SE, mean ± standard errors.

**Table 2:** Socio-demographic characteristics in subjects according to dynapenia status by sex.

Factors	Categories	Men			Women		
		Dynapenia (*n* = 557)	Normal (*n* = 9177)	*p*	Dynapenia (*n* = 1078)	Normal (*n* = 10,138)	*p*
		M or %	M or %		M or %	M or %	
Age	Total	24.04 ± 0.19	42.34 ± 0.10	<0.001	15.35 ± 0.08	25.58 ± 0.06	<0.001
Education	Low	70.8	40.8	<0.0001	65.6	47.6	<0.0001
	High	29.2	59.2		34.4	52.4	
Marital status	with	46.1	62.6	<0.001	51.1	63.8	<0.001
	without	53.9	37.4		48.9	36.2	
Individual income	Q1 (Lowest)	31.6	23.4	<0.001	29.5	24.3	0.008
	Q2	24.3	25.4		24.1	25	
	Q3	21.9	25.9		22.1	25.5	
	Q4 (Highest)	22.2	25.4		24.3	25.2	

Q1, quartile 1; Q2, quartile2; Q3, quartile3; Q4, quartile 4.

**Table 3:** Health- and disease-related characteristics in subjects according to dynapenia status by sex.

Factors	Categories	Men			Women		
		Dynapenia	Normal	*p*	Dynapenia	Normal	*p*
		M or %	M or %		M or %	M or %	
Grip strength (kg)		23.76 ± 0.16	42.12 ± 0.10	<0.0001	15.37 ± 0.08	25.53 ± 0.06	<0.0001
Height (cm)		165.22 ± 1.39	172.26 ± 0.08	<0.001	154.95 ± 0.26	159.51 ± 0.07	<0.001
Weight (kg)		62.11 ± 0.63	72.90 ± 0.15	<0.001	54.91 ± 0.35	58.24 ± 0.11	<0.001
BMI (kg/m^2^)	Index	22.66 ± 0.19	24.53 ± 0.04	<0.001	22.88 ± 0.14	22.89 ± 0.04	0.902
	Low	15.1	2.7	<0.0001	10.1	7.1	0.007
	Normal	58	56.4		64.5	69.3	
	Overweight	21.5	34.7		21.3	19.3	
	Obesity	5.4	6.2		4.1	4.3	
Blood pressure (mmHg)	Systolic	116.86 ± 0.66	118.87 ± 0.18	0.003	115.92 ± 0.67	111.69 ± 0.18	<0.001
	Diastolic	71.69 ± 0.46	78.24 ± 0.13	<0.001	71.95 ± 0.33	72.77 ± 0.12	0.018
	Hypertension	23.5	32	<0.001	24.8	16.6	<0.001
Fasting glucose (mg/dL)	Index	101.51 ± 1.21	100.94 ± 0.28	0.645	99.82 ± 0.84	94.95 ± 0.21	<0.001
	Diabetes	35.4	36.2	0.719	33	21.2	<0.001
TG	Index	123.29 ± 3.46	163.55 ± 1.81	<0.001	112.05 ± 2.20	105.44 ± 0.85	0.004
	High	28.9	39.3	<0.001	21.7	16.7	<0.001
HDL-cholesterol	Index	48.09 ± 0.48	47.84 ± 0.13	0.615	53.58 ± 0.44	56.18 ± 0.15	<0.001
	Low	26.3	24.3	0.301	41.7	33.5	<0.001
WC (cm)	Index	81.39 ± 0.55	85.78 ± 0.12	<0.001	77.94 ± 0.39	76.81 ± 0.13	0.005
	abdominal obesity	24.2	31.3	0.001	26.5	19.2	<0.001
Smoking status	current	23.6	37.8	<0.0001	4.2	6	0.023
	past	28.1	33.2		5.1	6.5	
	non	48.2	29		90.7	87.5	
Alcohol status	Yes	54.8	86.1	<0.0001	52.5	72.6	<0.0001
	No	45.2	13.9		47.5	27.4	
Aerobic exercise	TWT	247.91 ± 19.36	248.20 ± 4.67	0.988	199.96 ± 11.43	240.94 ± 4.16	<0.001
Resistance exercise	Never	80.1	68.3	<0.001	90.3	85	<0.001
	Mid	9.9	14.9		5.9	9.2	
	High	10	16.8		3.8	5.8	

**Table 4:** Risk factors for hand-grip-determined dynapenia in men.

Factors	Categories	Crude		Adjusted	
		OR (95% CI)	*p*	OR (95% CI)	*p*
Age	20s	1		1	
	30s	0.423 (0.256–0.699)	<0.001	0.394 (0.227–0.686)	0.001
	40s	0.444 (0.2269–0.733)	0.002	0.359 (0.201–0.644)	<0.001
	50s	0.675 (0.445–1.025)	0.065	0.498 (0.306–0.809)	0.005
	60s	1.641 (1.108–2.431)	0.014	1.136 (0.698–1.849)	0.61
	70s or older	7.177 (5.208–9.892)	<0.001	3.468 (2.307–5.213)	<0.001
Education	Low	3.512 (2.824–4.368)	<0.001	1.512 (1.155–1.979)	0.003
	High	1		1	
Marital status	with	0.510 (0.421–0.617)	<0.001	0.968 (0.714–1.314)	0.833
	without	1		1	
Individual income	Q1 (Lowest)	1.546 (1.211–1.975)	<0.001	1.373 (1.056–1.784)	0.018
	Q2	1.096 (0.841–1.428)	0.496	1.006 (0.754–1.341)	0.972
	Q3	0.970 (0.737–1.278)	0.831	0.877 (0.65–1.185)	0.391
	Q4 (Highest)	1		1	
BMI (kg/m^2^)	Low	5.442 (4.014–7.378)	<0.001	2.592 (1.785–3.764)	<0.001
	Normal	1		1	
	Overweight	0.602 (0.476–0.760)	<0.001	0.737 (0.535–1.016)	0.062
	Obesity	0.836 (0.511–1.367)	0.475	0.986 (0.534–1.82)	0.963
Blood pressure (mmHg)	Normal	1		1	
	Hypertension	0.652 (0.526–0.808)	<0.001	0.815 (0.644–1.032)	0.089
Fasting glucose (mg/dL)	Normal	1		1	
	Diabetes	0.967 (0.806–1.161)	0.719	1.112 (0.892–1.386)	0.348
TG	Normal	1		1	
	High	0.628 (0.517–0.762)	<0.001	1.044 (0.826–1.319)	0.723
HDL-C	Normal	1		1	
	Low	1.112 (0.909–1.359)	0.301	1.299 (1.015–1.661)	0.038
WC (cm)	Normal	1		1	
	abdominal obesity	0.702 (0.564–0.873)	0.001	1.014 (0.729–1.411)	0.936
Smoking status	current	0.376 (0.296–0.479)	<0.001	0.943 (0.690–1.288)	0.708
	past	0.510 (0.410–0.634)	<0.001	1.069 (0.805–1.419)	0.646
	non	1		1	
Alcohol status	Yes	0.196 (0.162–0.236)	<0.001	0.437 (0.348–0.548)	<0.001
	No	1		1	
Aerobic exercise	TWT	1.000 (0.997–1.003)	0.988	1.001 (0.998–1.004)	0.683
Resistance exercise	Never	1.984 (1.494–2.635)	<0.001	2.386 (1.771–3.214)	<0.001
	Mid	1.128 (0.739–1.723)	0.577	1.208 (0.766–1.903)	0.417
	High	1		1	

**Table 5:** Risk factors for hand-grip-determined dynapenia in women.

Factors	Categories	Crude		Adjusted	
		OR (95% CI)	*p*	OR (95% CI)	*p*
Age	20s	1		1	
	30s	0.697 (0.521–0.931)	0.014	0.623 (0.458–0.846)	0.002
	40s	0.708 (0.531–0.944)	0.019	0.586 (0.434–0.79)	<0.001
	50s	1.077 (0.809–1.433)	0.613	0.754 (0.536–1.06)	0.104
	60s	1.890 (1.418–2.519)	<0.001	1.089 (0.771–1.539)	0.63
	70s or older	4.181 (3.263–5.358)	<0.001	2.392 (1.751–3.267)	<0.001
Education	Low	2.105 (1.780–2.490)	<0.001	1.197 (0.966–1.484)	0.101
	High	1		1	
Marital status	with	0.595 (0.513–0.690)	<0.001	0.941 (0.782–1.133)	0.52
	without	1		1	
Individual income	Q1 (Lowest)	1.257 (1.033–1.529)	0.022	1.155 (0.944–1.413)	0.163
	Q2	0.998 (0.804–1.238)	0.982	0.966 (0.77–1.213)	0.763
	Q3	0.895 (0.727–1.103)	0.3	0.869 (0.699–1.08)	0.204
	Q4 (Highest)	1		1	
BMI (kg/m^2^)	Low	1.526 (1.156–2.016)	0.003	1.423 (1.055–1.919)	0.021
	Normal	1		1	
	Overweight	1.187 (0.995–1.415)	0.056	0.781 (0.627–0.974)	0.028
	Obesity	1.029 (0.726–1.458)	0.873	0.594 (0.405–0.871)	0.008
Blood pressure (mmHg)	Normal	1		1	
	Hypertension	1.663 (1.412–1.960)	<0.001	1.187 (0.994–1.418)	0.06
Fasting glucose (mg/dL)	Normal	1		1	
	Diabetes	1.833 (1.565–2.146)	<0.001	1.476 (1.242–1.754)	<0.001
TG	Normal	1		1	
	High	1.388 (1.177–1.638)	<0.001	1.14 (0.939–1.384)	0.187
HDL-C	Normal	1		1	
	Low	1.419 (1.235–1.630)	<0.001	1.084 (0.93–1.264)	0.305
WC (cm)	Normal	1		1	
	abdominal obesity	1.521 (1.296–1.785)	<0.001	1.419 (1.146–1.758)	0.001
Smoking status	current	0.671 (0.484–0.931)	0.017	0.861 (0.608–1.218)	0.395
	past	0.759 (0.540–1.066)	0.111	1.07 (0.75–1.529)	0.71
	non	1		1	
Alcohol status	Yes	0.417 (0.358–0.485)	<0.001	0.655 (0.553–0.776)	<0.001
	No	1		1	
Aerobic exercise	TWT	0.995 (0.991–0.999)	0.011	0.996 (0.993–0.999)	0.02
Resistance exercise	Never	1.621 (1.112–2.364)	0.012	1.375 (0.931–2.031)	0.109
	Mid	0.978 (0.616–1.552)	0.924	0.976 (0.606–1.571)	0.917
	High	1		1	

## Data Availability

All data were anonymized and can be downloaded from the website (https://knhanes.kdca.go.kr/knhanes, accessed on 10 May 2024).
